# A new analysis of heart rate variability in the assessment of fetal parasympathetic activity: An experimental study in a fetal sheep model

**DOI:** 10.1371/journal.pone.0180653

**Published:** 2017-07-10

**Authors:** C. Garabedian, C. Champion, E. Servan-Schreiber, L. Butruille, E. Aubry, D. Sharma, R. Logier, P. Deruelle, L. Storme, V. Houfflin-Debarge, J. De Jonckheere

**Affiliations:** 1 Univ. Lille, EA 4489 – Perinatal Environment and Health, Lille, France; 2 CHU Lille, Department of Obstetrics, Lille, France; 3 CHU Lille, Department of Pediatric Surgery, Lille, France; 4 CHU Lille, CIC-IT 1403, MRRC, Lille, France; 5 CHU Lille, Department of Neonatology, Lille, France; University of Minnesota, UNITED STATES

## Abstract

Analysis of heart rate variability (HRV) is a recognized tool in the assessment of autonomic nervous system (ANS) activity. Indeed, both time and spectral analysis techniques enable us to obtain indexes that are related to the way the ANS regulates the heart rate. However, these techniques are limited in terms of the lack of thresholds of the numerical indexes, which is primarily due to high inter-subject variability. We proposed a new fetal HRV analysis method related to the parasympathetic activity of the ANS. The aim of this study was to evaluate the performance of our method compared to commonly used HRV analysis, with regard to i) the ability to detect changes in ANS activity and ii) inter-subject variability. This study was performed in seven sheep fetuses. In order to evaluate the sensitivity and specificity of our index in evaluating parasympathetic activity, we directly administered 2.5 mg intravenous atropine, to inhibit parasympathetic tone, and 5 mg propranolol to block sympathetic activity. Our index, as well as time analysis (root mean square of the successive differences; RMSSD) and spectral analysis (high frequency (HF) and low frequency (LF) spectral components obtained via fast Fourier transform), were measured before and after injection. Inter-subject variability was estimated by the coefficient of variance (%CV). In order to evaluate the ability of HRV parameters to detect fetal parasympathetic decrease, we also estimated the effect size for each HRV parameter before and after injections. As expected, our index, the HF spectral component, and the RMSSD were reduced after the atropine injection. Moreover, our index presented a higher effect size. The %CV was far lower for our index than for RMSSD, HF, and LF. Although LF decreased after propranolol administration, fetal stress index, RMSSD, and HF were not significantly different, confirming the fact that those indexes are specific to the parasympathetic nervous system. In conclusion, our method appeared to be effective in detecting parasympathetic inhibition. Moreover, inter-subject variability was much lower, and effect size higher, with our method compared to other HRV analysis methods.

## Introduction

Under hypoxia, the fetus develops several coping mechanisms, including an increase in O_2_ extraction by tissues and the redistribution of blood flow toward the vital organs. These adaptive mechanisms are regulated by the autonomic nervous system (ANS) via sympathetic and parasympathetic activities. The ANS is highly sensitive to hypoxia and acidosis, through various well-described mechanisms, including the stimulation of chemoreceptors and baroreceptors, or the direct actions of hypoxia and hypercapnia on the brainstem [1]. Therefore, an analysis of ANS activity could be an efficient tool in evaluating fetal well-being [2].

Analysis of heart rate variability (HRV) is a recognized non-invasive tool that is used to assess ANS regulation, and a variety of time and spectral analysis methods have been proposed to evaluate fetal HRV [3]. One of the main methods used to perform time analysis is the root mean square of the successive differences (RMSSD) of R—R intervals in the electrocardiogram (ECG) [4], which corresponds to an estimation of the short-term variations analysis. The spectral analysis of the HRV can be quantified by the use of signal processing algorithms allowing us to obtain the heart rate frequency components of the following frequency ranges: very low frequency (VLF), a range of between 0 and 0.04 Hz; low frequency (LF), a range of between 0.04 and 0.15 Hz; and high frequency (HF), above 0.15 Hz [3]. The VLF range is related to thermoregulatory and endocrine activities; the LF range is related to the modulation of both the sympathetic and parasympathetic nervous systems, and is dependent on the feedback activity of baroreceptors; the HF range is centered on the respiratory frequency and is related to parasympathetic modulation only [5]. Using spectral analysis of fetal HRV, several previous studies have shown significant changes in the HF range in hypoxic fetuses, suggesting variations in parasympathetic activity [6–8].

However, existing signal processing methods are limited in terms of the lack of thresholds of the numerical indexes, which is primarily due to high inter-subject variability. This lack of thresholds to enable the prediction of ANS changes means that the methods cannot be used in obstetrical clinical practice.

We proposed a new original method that allowed us to obtain a numerical index relative to the parasympathetic activity of the ANS. Although most of the published methods are based on spectral or time analysis of HRV, this numerical index uses spectral analysis to filter the signal in order to keep only HF oscillations, and then computes the magnitude of these oscillations in the time domain.

The aim of this study was to evaluate the performance of our method compared to commonly used HRV analysis, regarding i)the ability to detect the parasympathetic activity of the fetus and ii) inter-subject variability.

## Materials and methods

### Surgical preparation

Near-term pregnant Charmoise sheep (INRA, Leudeville, France) with a gestational age of 123 +/- 2 days, term = 145 days, underwent the surgical procedure. The anesthetic and surgical technique protocols followed those previously described by our team or in previous studies [9,10]. The sheep fasted for 24 h before general anesthesia and surgery. Before surgery, sheep were placed in a supine position, anesthetized with an intravenous injection of xylazine (Sedaxylan^®^, CEVA Santé Animale, France), intubated, and maintained with isoflurane 2% (Aerrane^®^, Baxter, France). The uterus was exteriorized via a maternal midline laparotomy. Following hysterotomy, a 4Fr diameter catheter (Arrow^®^, USA) was inserted into the femoral artery and vein until reaching the abdominal aorta and the inferior vena cava, respectively, through the femoral approach. Four precordial electrodes (MYWIRE 10^®^, MAQUET, Germany) were placed subcutaneously in order to detect an ECG signal. A 5F5-diameter catheter (Arrow^®^, USA) was placed into the amniotic cavity for measuring baseline pressure (intra-amniotic pressure; IAP). The fetal arterial catheter and intra-amniotic catheter were connected to pressure sensors (Pressure Monitoring Kit^®^, Baxter, France) that were connected to a blood pressure monitor (monitor Merlin, Hewlett Packard, Palo Alto, CA, USA). The mean arterial pressure (MAP) was measured from the blood pressure phasic signal and referenced to the IAP (calculated MAP = observed MAP—observed IAP). All hemodynamic data were recorded with the Physiotrace^®^ data acquisition board (Estaris monitoring, Lille, France).

### Experimental procedure

The experiments began only after the sheep had had 48 hours of rest. Prior to injection (one per 24h per animal), an initial 30-minute period, called the stability period, was recorded. Fetal hemodynamic parameters (heart rate, blood pressure, and intra-amniotic pressure) were recorded every 5 minutes for the duration of the experiment (30 min). Recording of the electrocardiographic signal was continuous over the same period. Fetal arterial and venous blood gases were analyzed at the beginning and end of each protocol to ensure that the animals were healthy and representative of the model.

Two drugs were tested according to their effects on the autonomic nervous system: 2.5 mg atropine (Atropine, Aguettant, France), to evaluate the effect of parasympathetic nervous system inhibition, and 5 mg propranolol (Karnodyl^®^, Primius Lab, France), a non-selective beta-blocker with a sympatholytic effect [11], were intravenously injected into the femoral vein.

Following the recovery period, the ewe and the fetus were sacrificed via an injection of embutramide(T61^®^, Intervet, France).

### ECG signal preprocessing

The fetal ECG signal was recorded using the Physiotrace^™^ data acquisition board (Physiotrace^™^, Estaris Monitoring, Lille, France) [12], and ECG analysis was conducted off-line using an automatic R wave detection algorithm. R waves that missed detection, false detection, and ectopic beats were identified and replaced by a linear interpolation using a specific artifact removal algorithm [13]. As recommended, the resulting R-R series were re-sampled at an 8 Hz frequency using a linear interpolation algorithm [3].

### Fetal stress index analysis

Jeanne et al. studied HRV in a simulated environment and confirmed that HRV spectral analysis was sensitive to i) the basal heart rate, ii) the heart rate variations magnitudes and iii) the heart rate oscillations frequency [14]. In order to reduce inter-subject variability we therefore developed the algorithm described bellow;

The re-sampled R-R series was isolated in a 64-second moving window. In a first step, the signal is mean centered to eliminate the influence of the basal heart rate value.

The mean value (M) is computed as:

$$\text{M}=\frac{\text{1}}{\text{N}}\sum_{\text{i}=\text{1}}^{\text{N}}{\text{(RR}}_{\text{i}}\text{)},$$

Where RR_i_ represents the RR samples values and N represent the number of samples in the window. M is then subtracted from each sample of the window as: RR’_i_ = (RR_i_−M).

In a 2nd step, the signal is normalized to reduce the influence of the heart rate variations magnitude. The norm value (S) is then calculated as:

$$\text{S}=\sqrt{\sum_{\text{i}=\text{1}}^{\text{N}}{\left({\text{RR'}}_{\text{i}}\right)}^{\text{2}}}.$$

and each RR’_I_ is divided by S: RR”_i_ = RR’_i_ / S.

The normalized RR” series is then high-pass filtered above 0.15 Hz, using a 4 coefficient Daubeuchies wavelet-based filter in order to keep only high frequency oscillations.

In order to eliminate the influence on the resulting oscillations frequency, we estimate the area under the envelope of the mean, normalized and filtered R-R series. The local maximum and minimum are detected via this filtered signal, and the lower and upper envelopes were plotted.

Jeanne et al. demonstrated that this method was not sensitive to basal heart rate, heart rate variations magnitude or heart rate HF oscillations frequency while keeping a strong correlation with commonly used HRV spectral measurements [14].

**Fig 1:**
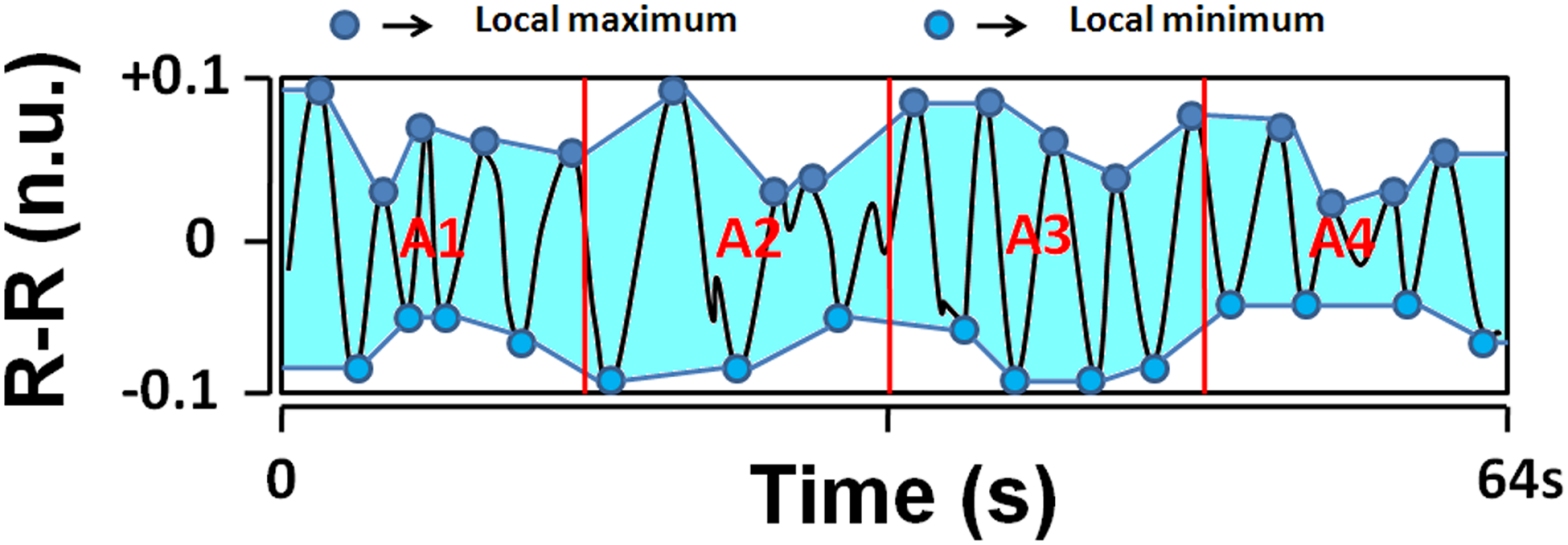
A normalized and filtered RR series (black curve, in normalized unit). Areas A1, A2, A3, and A4 are computed between the lower and upper limits (area in grey). The lowest area (AUCmin) is then selected (A4) and Fetal Stress Index is computed.

In order to increase the time-sensitivity of the method and to allow detecting rapid transient drops in the parasympathetic activity, we divided the envelope in four 16-seconds sub-areas A1, A2, A3, and A4 (Fig 1). AUC_min_ was determined as the minimum value of A1, A2, A3, and A4 and fetal stress index (FSI) was defined as: FSI = 100 x (5.1 x AUC_min_ + 1.2) / 12.8, where *a* = 5.1 and *b* = 1.2 were empirically determined in a dataset of more than 200 R-R records analysis in order to maintain good coherence between the visual aspect of the filtered R-R series (Fig 1) and a quantitative measurement ranging from 0 to 100.

Continuous computation of the FSI was assumed by sliding the moving window with a 1 second moving period. Mean values of the FSI were analyzed over two predefined 5-minute periods: 1) between 10 and 5 minutes before atropine or propranolol administration, and 2) between 5 and 10 minutes after atropine or propranolol administration.

The period around the time of injection was not analyzed in order to avoid any mathematical artifacts due to a rapid increase or decrease of fetal heart rate.

### Time and spectral analysis

HF and LF spectral components were computed using a Fast Fourier Transform (FFT) performed in a 256-seconds moving window with an 8-Hz sampling frequency (3). The moving window was mean-centered and passed through a Bartlett ponderation window prior to the FFT computation. Continuous computation of HF and LF was assumed by sliding the moving window with a 1 second moving period. Mean values of HF and LF were analyzed over the two previously defined 5-minute periods. Normalized HF was also computing as HF/ (HF+LF). RMSSD time analysis was also computed for these two periods.

### Ethics

All applicable international, national, and/or institutional guidelines for the care and use of animals were followed. The anesthesia, surgery, and experimentation protocols were performed as recommended by the Ministry of Higher Education and Research, and the studywas approved by the Animal Experimentation Ethics Committee (APAFiS #5160).

### Statistical analysis

Given the small sample size, a Wilcoxon non parametrical test was used to compare data obtained before and after injections. Statistical significance was defined asp< 0.05. In order to evaluate the ability of HRV parameters with regard to differentiating the periods before and after injection, we also estimated the effect size (i.e. the magnitude of the difference) for each parameter, before and after drug injection.

Effect size was evaluated through the Cohen’s d coefficient, defined as:

$$\text{d}=\frac{\bar{D}}{\sqrt{\frac{SS_{D}}{n-1}}}$$

Where $\bar{D}$ represents the mean difference between data before and after drug injection and SS_D_represents the sum of squared deviations (i.e. the sum of scores of deviations from the mean difference scores). The inter-subject variability was estimated by computing the coefficient of variance (%CV), defined as: %CV = 100*SD/M, where SD represents the standard deviation and M represents the mean value. %CV was computed for FSI, LF, HF, and RMSSD prior to atropine and propranolol injection. Hemodynamic, gazometric, and HRV parameters were expressed as median values (1^st^ quartile– 3^rd^ quartile). We used Statistical Package for the Social Sciences, version 20.0, software (IBM, Armonk, NY, USA).

## Results

Seven near-term pregnant sheep were chronically instrumented.

### Atropine

Seven experimental procedures were performed. Table 1 shows the data obtained before and after atropine injection. Following direct intravenous injection of atropine, HR significantly increased from 156(140.2–168.2) to 190(181.2–199.2) bpm (p = 0.028), while the mean arterial blood pressure remained the same (47(45–59)–52(49–59), p = 0.225); HF significantly decreased from 52.9(37.2–75) to 25.4(21.1–39.1) (p = 0.043); RMSSD significantly decreased from 8.7(6.3–9.2) to 4(3–6.5) (p = 0.018); HF/(HF+LF) significantly decreased from 0.68(0.61–0.78) to 0.53(0.47–0.65) (p = 0.018); and FSI significantly decreased from 57.3(52.4–61.2) to 40.4(36–46.2) (p = 0.018). %CV was important for HF, LF and RMSSD (respectively 33.4%, 35.2% and 49.3% before injection). %CV was far lower for HF(HF+LF) (%CV = 11.6 before injection) and the FSI (%CV = 9.9 before injection). Although the RMSSD, HF(HF+LF) and FSI p values were similar (p = 0.018), the effect size was greater for FSI. Indeed, as Gail *et al*. found, HF, HF/(HF+LF) and RMSSD were associated with a large effect size (>0.8) and FSI with a very large effect size (>1.3) [15].

**Table 1:** Spectral analysis (HF, LF and normalized HF), RMSSD and fetal stress index values before and after direct intravenous administration of 2.5 mg atropine. The results are presented as median values (1^st^–3^rd^ quartiles). A Wilcoxon test was used for analysis. Statistical significance was set as p <0.05.

	Before	%CV before	After	%CV after	p	Effect size
**HF**	52.9(37.2–75)	33.4	25.4(21.1–39.1)	30.2	0.043	1.13
**LF**	22.3(16–31.1)	35.2	18.1(14–41.8)	51.1	0.398	0.35
**HF/(HF+LF)**	0.68(0.61–0.78)	11.6	0.53(0.47–0.65)	18.3	0.018	0.92
**RMSSD**	8.7(6.3–9.2)	49.3	4(3–6.5)	37.6	0.018	1.13
**FSI**	57.3(52.4–61.2)	9.9	40.4(36–46.2)	17.3	0.018	1.49

**Fig 2:**
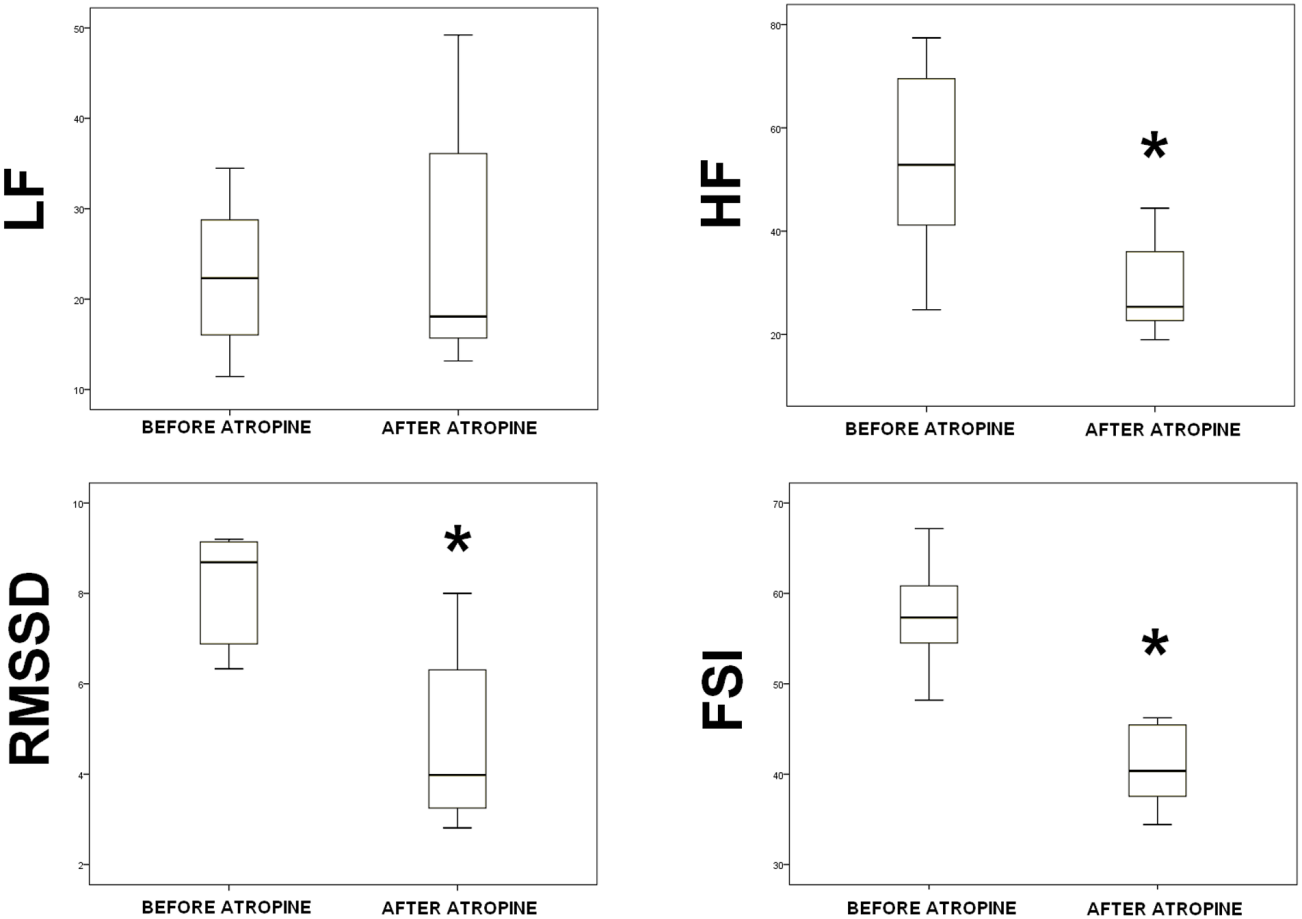
High frequency, low frequency, root mean square of the successive differences and fetal stress index before and after administration of 2.5 mg atropine. * → p<0.05 versus before atropine injection.

HF = high frequency; LF = low frequency,

RMSSD = root mean square of the successive differences;

Fig 2 shows the box plot of HF, LF, RMSSD and FSI variations before and after direct intravenous atropine administration.

### Propranolol

Seven experimental procedures were performed; one was excluded due to poor ECG quality. Table 2 shows the data obtained before and after propranolol injection. Following propranolol injection, ABP remained stable (40(36–44) vs (42(39–45), p = 0.092); HR decreased (161(155–165) vs 147(143–155), p = 0.046, as expected; HF, HF/(HF+LF), RMSSD and FSI did not vary significantly; and LF was lower (28(20.9–33.6) vs 12.7(6–16.2), p = 0.046).

**Table 2:** Spectral analysis (HF, LF and normalized HF), RMSSD and fetal stress index values before and after direct intravenous administration of propranolol. The results are presented as median values (1^st^–3^rd^ quartiles). A Wilcoxon test was used for analysis. Statistical significance was set as p <0.05.

	Before	%CV before	After	%CV after	p	Effect size
**HF**	52.3(34.8–78.1)	53.5	25.4(17.5–42.6)	43.1	0.075	0.81
**LF**	28(20.9–33.6)	35.4	12.7(6–16.2)	51.8	0.046	1.25
**HF/(HF+LF)**	0.66(0.64–0.76)	9.22	0.70(0.65–0.79)	10.9	0.345	0.38
**RMSSD**	6.7(3.5–13.5)	73.6	6.4(4.1–18.6)	91.7	0.917	0.39
**FSI**	56.5(48.25–59.25)	10.9	61.5(51–76)	21.9	0.599	0.45

HF = high frequency; LF = low frequency,

RMSSD = root mean square of the successive differences;

Gazometric parameters were comparable before and after the two protocols (Table 3).

**Table 3:** Blood gases before and after injection of atropine and propranolol, respectively. The results are presented as median values (1^st^–3^rd^ quartiles).

	Atropine			Propranolol		
	Before	After	p	Before	After	p
pH	7.39 (7.37–7.41)	7.40 (7.38–7.41)	0.39	7.38(7.26–7.40)	7.37(7.27–7.39)	0.22
pO2(mmHg)	15,0 (15.0–17.0)	16,00 (15.0–16.0)	0.26	16.0 (14.5–17.5)	16.0 (14.7–17.2)	1
pCO2(mmHg)	45.1 (42.5–47.9)	46.5(41.4–47.1)	0.87	41.0(39.3–45.7)	40.7(39.1–43.9)	0.67
Lactates(mmol/L)	1.2(1.1–1.3)	1.2(1.1–1.3)	0.25	1.1(1.0–1.5)	1.1(0.5–1.4)	0.34

## Discussion

A computerized analysis of FHR variability could provide promising markers of fetal distress, monitored via analysis of the ANS [6,16]. We developed an original HRV analysis algorithm toobtain a numerical index relative to the parasympathetic activity of the ANS. We hypothesized that this algorithm could be adapted for fetal HRV analysis in order to obtain an FSI related to fetal parasympathetic activity, and we compared this to common methods of HRV analysis.

In order to evaluate the effect of inhibition of the parasympathetic nervous system on HRV, we used direct administration of 2.5 mg intravenous atropine, which is often used in this manner [4,11]. Frasch et al. observed an FHR increase after atropine injection in a sheep model (181 vs 236 bpm, p < .05) [4]. Moreover, the spectralpower of the RMSSD and HF bands decreased, suggesting a decrease in vagal modulationof FHR. The spectral power of the LF bandincreased, suggesting vagal-mediated disinhibition ofsympathetic modulation of FHR (4). In our study, the FSI, HF, HF/(HF+LF), and RMMSD dropped after the atropine injection, as expected, indicating that the FSI is highly related to parasympathetic activity.

We then evaluated the inhibition of the sympathetic system by the use of the beta-blocker, propranolol; Frasch et al. had previously described the blocking effect of this system via propranolol use in seven sheep [11]. As expected, we did not observe any change in the FSI, RMMSD, and HF following propranolol administration. LF decreased, suggesting vagal blockade. Thus, these experimental results, correlated with the findings of previous studies, allowed us to confirm the specificity of our index for parasympathetic tone.

The interest in HRV in the present study remains in the fact that it could be a new tool to predict fetal acidosis during labor [2]. In a model of repeated cord occlusion in sheep, an increase of RMSSD was observed in association with FHR decelerations, reflecting initial vagal activation during fetal asphyxia, and the authors concluded that RMSSD mayaccurately identify hypoxic fetuses at an early stage [4]. Chung et al.investigated HRV at very low, low, and high frequencies during the last 2 hours before delivery [8], and observed a significant decrease in VLF, LF, and HF in acidotic fetuses. Siiraet al. also studied FHR variability during the last hour of labor according to the pH at birth [16]. Before delivery, they observed a significant decrease in HF variations in the acidotic group. Van Laar et al. found that acidotic fetuses had a significantly decreased in HF/(HF+LF) during the last 30 minutes of labor, compared with non-acidotic fetuses [6]. In all these studies, authors observed a drop in parasympathetic activity, as evaluated by the HF spectral component in acidotic fetuses. However, non-routine clinical use is proposed due to the lack of thresholds allowing the prediction of ANS changes related to fetal acidosis. In the present study, we compared common HRV analysis methods to the FSI and we showed that the FSI was associated with a higher effect size, showing that this parameter presented a higher power to detect parasympathetic inhibition. This very large effect size could help in the determination of thresholds for fetal acidosis prediction. Moreover, we compared dispersion of the data defined as the coefficient of variance %CV. The results highlightedthe fact that inter-subject variability was far lower with the FSI than with spectral analysis (HF and LF) and RMSSD.

This experimental model is associated with some limitations. The surgery performed to position electrodes and catheters may induce fetal pain, which is known to be responsible for a decrease in parasympathetic activity. However, all animals underwent the same surgical procedure, and a rest period of 48 hours was observed between surgery and experiment.

## Conclusion

Although most of the published methods were based on a spectral or time analysis of HRV, our computerized index results from a filtering method, followed by a continuous analysis of the filtered signal magnitude in the time domain. In this study, we demonstrated that the FSI was associated with a lower inter-subject variability and a higher effect size, suggesting that this index could better detect a parasympathetic decrease than commonly used HRV analysis. However, our results are preliminary and must be confirmed in a genuine clinical context (i.e. fetal acidosis prediction).

## Supporting information

S1 File RMSSD, spectral analysis and FSI datas. (PDF)
