# Supplementary material for: A new analysis of heart rate variability in the assessment of fetal parasympathetic activity: An experimental study in a fetal sheep model
Source: PLoS One. 2017 Jul 10;12(7):e0180653. doi: 10.1371/journal.pone.0180653 (PMC5503275; doi:10.1371/journal.pone.0180653)
Supplement: S1 File — (PDF) [file pone.0180653.s001.pdf]

Atropine

Manip	LF_t1	HF_t1	Hfnu_t1	RMSSD_t1	FSI_t1	LF_t2
1	16,061	45,138	0,73756107	9,07989231	56,6494293	18,076
2	22,304	77,416	0,77633373	9,2002169	60,4394073	13,156
3	15,949	75,463	0,82552619	21,2056727	52,3851089	13,726
4	31,146	64,045	0,6728052	8,68842459	48,1908035	41,762
5	11,444	24,792	0,68418148	6,34792845	67,165184	17,382
6	34,483	52,862	0,60520923	7,41513345	61,2293396	30,468
7	26,394	37,219	0,58508481	6,33190057	57,3366737	49,229

Propranolol

Manip	LF_T1	HF_T1	HFnu_T1	RMSSD_T1	FSI_T1	LF_T2
1	7,467	23,75	0,76080341	3,58887169	60	11,646
2	32,356	63,022	0,66076034	7,59670981	56	21,869
3	26,799	38,435	0,58918662	3,39411255	50	13,823
4	25,405	48,524	0,65635948	22,4273642	43	7,233
5	29,233	56,156	0,65764911	5,73643327	59	14,297
6	37,494	123,497	0,76710499	10,5198226	57	2,153

HF_t2	Hfnu_t2	RMSSD_t2	FSI_t2
21,104	0,53864216	3,98395475	34,4255486
24,238	0,64817885	3,04284876	35,960331
32,94	0,70586723	7,75899068	39,1351852
25,363	0,3778473	3,45432835	46,2421532
18,993	0,52214433	2,81208133	40,3662949
39,052	0,56173763	6,53986184	58,048687
44,455	0,47452073	6,07817292	44,665741

HF_T2	HFnu_T2	RSMSD_T2	FSI_T2
19,048	0,62057731	3,3901819	79
41,479	0,65477995	6,3880096	55
45,929	0,76866046	13,854963	67
19,09	0,72522129	32,8828223	75
31,688	0,68909427	4,31	39
13,038	0,85827134	6,33298245	56
